# UV-Cured Highly Crosslinked Polyurethane Acrylate to Serve as a Barrier against Chemical Warfare Agent Simulants

**DOI:** 10.3390/polym16111578

**Published:** 2024-06-02

**Authors:** Xucong Chen, Linjing Xiao, Hong Li, Yan Cui, Guiyou Wang

**Affiliations:** 1 Shanghai Key Laboratory of Advanced Polymeric Materials, School of Materials Science and Engineering, East China University of Science and Technology, Shanghai 200237, China; y82210256@mail.ecust.edu.cn; 2 Shanghai Key Laboratory of Electrical Insulation and Thermal Aging, School of Chemistry and Chemical Engineering, Shanghai Jiao Tong University, Shanghai 200240, China; lynn0822@sjtu.edu.cn; 3 State Key Laboratory of NBC Protection for Civilian, Beijing 102205, China

**Keywords:** polyurethane acrylate, UV curing, chemical warfare agents, barrier coating

## Abstract

Ultraviolet (UV) curing is an efficient and environmentally friendly curing method. In this paper, UV-cured polyurethane acrylates (PUAs) were investigated as potential military coatings to serve as barriers against chemical warfare agents (CWAs). Seven UV-cured PUA coatings were formulated utilizing hydroxyethyl methacrylate-capped hexamethylene diisocyanate trimer (HEMA-Htri) and trimethylolpropane triacrylate-capped polycarbonate prepolymer (PETA-PCDL) as the PUA monomers. Isobornyl acrylate (IBOA) and triethyleneglycol divinyl ether (DVE-3) were employed as reactive diluents. Gas chromatography was utilized to investigate the constitutive relationships between the structures of the PUA coatings and their protective properties against simulant agents for CWAs, including dimethyl methylphosphonate (DMMP), a nerve agent simulant, and 2-chloroethyl ethyl sulfide (CEES), a mustard simulant. The glass transition temperature (*T_g_*) and crosslinking density (*υ_e_*) of PUAs were found to be crucial factors affecting their ability to serve as barriers against CWAs. The incorporation of IBOA units led to enhanced *T_g_* and barrier performance of the PUAs, resulting in a DMMP retention of less than 0.5% and nearly 0 retention of CEES. However, an excessive introduction of polycarbonate chains decreased the *υ_e_* and barrier performance of the PUAs. These findings may offer valuable insights for enhancing the protection of UV-cured PU coatings against CWAs.

## 1. Introduction

In military composite coating systems [1,2,3,4,5,6], both high crosslinking to resist chemical agents and high strength to adapt to special application scenarios are required. Polyurethane (PU) coatings, which are generally used at the very surface as a topcoat [1,5,6], face the challenge of both exposure to chemical warfare agents (CWAs) and the durability of the coating. CWAs are highly toxic chemicals that can adsorb and permeate painted coatings, resulting in equipment damage and secondary contamination [1,7,8,9]. PU’s resistance to adsorption and permeation of some polar agents or CWAs is poor [1,4,10,11]. Levine [4] et al. found that increasing the relative amounts of isocyanates and hydroxyl groups (R value) in waterborne polyurethane coatings improved the permeation resistance of Dimethyl methylphosphonate (DMMP), which suggests that increasing crosslinking is a possible way to improve the resistance to CWAs. Our previous study found that the crosslinking and chemical structure of the coating impacts the coating’s resistance to CWAs [12,13]. Ultraviolet (UV)-cured PU coatings have excellent characteristics such as fast curing speed, environmentally friendly, high hardness, high transparency, etc. [14,15,16,17,18,19,20,21,22,23]. Due to the low molecular weight of the UV-curing monomers used, UV-cured PU coatings have higher crosslinking density and also have the potential to be applied in the CWA resistance coating.

Typically, chemically and structurally similar CWA simulants are often used in experiments in place of genuine CWAs with toxic properties. DMMP, a simulant with the same phosphate ester (P=O) structure as organophosphorus CWAs and with hydrogen-bonded donors, was used in experiments as a simulant of G or V series nerve agents [1,12,24]. 2-Chloroethyl ethyl sulfide (CEES) is a “half-mustard gas” and is often used as a simulant for sulfur mustard (HD) [1,25,26]. Due to the high reactivity of carbon–chlorine bonds (C-Cl), sulfonium groups (hydrogen bond donors) are formed and react with hydrogen bond acceptors [27]. Different CWAs, due to their different properties, should have different resistance coating types. There are also few reports on the effect of UV-cured coating structures on the chemical resistance of different agents.

Herein, UV-curable polyurethane acrylates (PUAs) were prepared for use as potential barriers against CWAs for military coatings of metal surfaces on vehicles or weapons. The PUA monomers were hexamethylene diisocyanate trimer capped with hydroxyethyl methacrylate (HEMA-Htri) and polycarbonate diol capped with hydroxymethylpropane triacrylate and isophorone diisocyanate (PETA-PCDL). The hexamethylene diisocyanate trimer possesses trifunctionality, whereas the polycarbonate diol contributes to the toughness of the polymer. The reactive diluents were isobornyl acrylate (IBOA) and triethyleneglycol divinyl ether (DVE-3). IBOA enhances the *T_g_* of the polymer due to the site barrier effect, while DVE-3 improves the flexibility of the polymer owing to the presence of three ether bonds. Seven UV-cured PUA coatings were prepared. The PUA monomers were characterized by Fourier transform infrared spectroscopy (FTIR), ^1^H nuclear magnetic resonance (^1^H NMR), and differential scanning calorimetry (DSC). The PUA coatings were characterized by FTIR, and their UV-cured chemical structure was characterized. The thermomechanical properties of PUAs were characterized by dynamic mechanical thermal analysis (DMA) and universal tensile machine. The chemical protection properties were assessed by measuring the retention of DMMP and CEES in the PUA coatings by gas chromatography (GC) to determine the barrier performance of PUAs with different structures. The introduction of IBOA to PUAs demonstrated a significant increase in the *T_g_* of PUAs, leading to improved DMMP and CEES resistance. These findings offer constructive suggestions for enhancing the protection of UV-cured PU coatings against CWAs.

## 2. Experimental Section

### 2.1. Materials

2-Hydroxyethyl methacrylate (HEMA, 97%), isophorone diisocyanate (IPDI, 99%), 1,1,1-tris(Acryloyloxymethyl)propane (PETA, 95%), ethyl acetate (EA, anhydrous grade, 99.8%), dibutyltin dilaurate (DBTDL, 95%), dichloromethane (DCM, anhydrous grade, 99.9%), 1-hydroxycyclohexyl phenyl ketone (184, UV initiator, 98%), phenyl (2,4,6-trimethylbenzoyl) ethyl phosphite (TPO-L, UV initiator, 98%), N-nitroso-N-phenylaluminium hydroxylamine aluminum salt (polymerization inhibitor, 98%), and trithyleneglycol dvinyl ether (DVE-3, 98%) were obtained from Shanghai Titan Technology Co., Ltd. (Shanghai, China). Hexamethylene diisocyanate trimer (HDI trimer, WANNATE HT100, 100% solid content) was obtained from Wanhua Chemical Group Co., Ltd. (Yantai, China). Dimethyl methylphosphonate (DMMP, 98%, industrial grade) was purchased from Jinbang Chemical Co., Ltd. (Jinan, China). 2-Chloroethyl sulfide (CEES, 97%) was from Macklin Reagent Co., Ltd. (Shanghai, China). Isobornyl acrylate (IBOA, 99%) was purchased from Shanghai Guangyi Chemical Co. Ltd. (Shanghai, China). All of these reagents were directly used as received without further purification. Polycarbonate diol (PCDL-1000, T5651) was purchased from Xuzhou Tacteasy New Material Co., Ltd. (Xuzhou, China). All of these reagents were directly used as received without further purification.

### 2.2. Synthesis of the PUA Monomer HEMA-Htri

**Figure 1:**
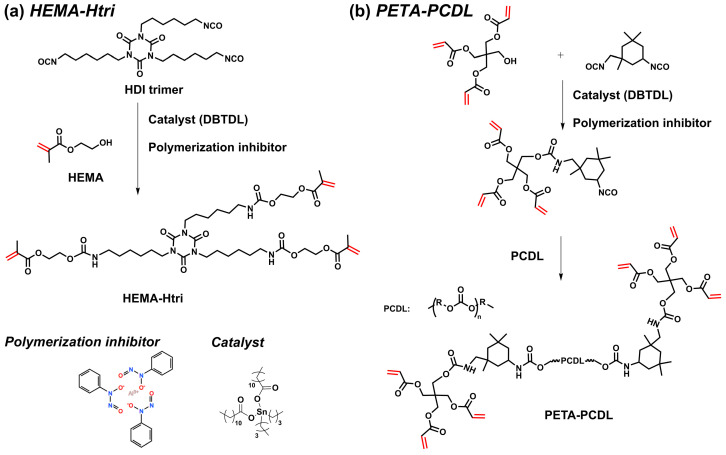
Synthetic route of the PUA monomer (**a**) HEMA-Htri and (**b**) PETA-PCDL.

HEMA (39.04 g, 0.3 mol), a polymerization inhibitor (0.1 wt.% of total resin), and EA (10 mL) were mixed well and added to a three-necked flask. HDI trimer (50.46 g, 0.1 mol), DBTDL (2 drops as a catalyst), and EA (20 mL) were mixed well and added to a dropping funnel. The mixture was stirred magnetically, and then the HDI trimer mixture was added dropwise to the flask under a nitrogen atmosphere. The reaction was performed at 60 °C for 4 h and then 80 °C for 0.5 h. The -NCO group in the solution was determined according to the toluene-dibutylamine method (ASTM D2572-19) [18,19]. The polymer solution was precipitated with petroleum ether and re-dissolved in ethyl acetate, and the purification of PUA monomer was repeated at least twice. Most of the solvent was removed by rotary evaporation at 60 °C, and then the polymer was dried under vacuum at 80 °C to obtain the purified PUA monomer HEMA-Htri. The synthetic routes of the HEMA-Htri are shown in Figure 1a, and their structure and properties were characterized by ^1^H NMR, FTIR, and DSC (Figures S1–S3).

### 2.3. Synthesis of the PUA Monomer PETA-PCDL

PETA-PCDL was synthesized in a similar way. PETA (29.83 g, 0.10 mol), IPDI (22.23 g, 0.10 mol), a polymerization inhibitor (0.1 wt.% of total resin), and EA (20 mL) were mixed well and added to a three-necked flask. Then, PCDL diol (50 g, 0.05 mol), DBTDL (2 extra drops), and EA (30 mL) were mixed well and added to a dropping funnel. The mixture was stirred magnetically, and then the PCDL diol mixture was added dropwise to the flask under a nitrogen atmosphere. The reaction was performed at 60 °C for 1 h and then 80 °C for 0.5 h. The -NCO group in the solution was determined according to the toluene-dibutylamine method (ASTM D2572-19) [18,19]. The polymer purification method is the same as in Section 2.2. The synthetic routes of the PETA-PCDL are shown in Figure 1b, and the structure and properties were characterized by ^1^H NMR, FTIR, and DSC (Figures S1–S3).

### 2.4. Preparation of the PUA Coatings

The UV initiators TPO-L and 184 were dissolved and mixed well with the reactive diluent (IBOA or DVE-3). The mixture was then combined with the PUA monomer (HEMA-Htri or PETA-PCDL), stirred thoroughly, vacuum defoamed for 10 min, and poured into polytetrafluoroethylene molds or scraped directly onto different substrates. PUA coatings were prepared using a coating rod with a thickness of 200 ± 20 µm. The specific formulations are shown in Table 1. The PUA coatings were UV-cured by irradiation with a 395 nm UV light (1000 W UV Curing Chamber) for 5 min, resulting in seven UV-cured PUAs. For PUA-Htri-DVE/IBOA*m*, “*m*” denotes that IBOA partially replaces DVE-3, with IBOA comprising “*m*” wt.% of the total resin. For PUA-Htri/PCDL*n*-DVE, “*n*” denotes that PETA-PCDL partially replaces HEMA-Htri, and PETA-PCDL comprises “*n*” wt.% of the total resin.

## 3. Characterization

### 3.1. Characterization of the Physical and Chemical Structure

The ^1^H Nuclear Magnetic Resonance (^1^H NMR) of the PUA monomer was performed using Bruker 500 NMR (AVANCE III HD 500), and the deuterated reagent used was DMSO-d6. Fourier infrared spectroscopy (FTIR) of the PUA coatings was characterized using Spectrum 100 from Perkin Elmer. PUA monomers were dropped onto potassium bromide tablets and measured by FTIR transmission mode. Dynamic FTIR was measured at regular intervals under UV light using an 8W circular UV light source. The viscosity of uncured PUA was carried out by using a rotational viscometer DV-II Pro from Brookfield, with a rotor model SC-29 and a speed of 5 rpm.

### 3.2. Characterization of Thermodynamic Properties

Dynamic mechanical analysis (DMA) was performed using a TA Discovery DMA 850 at a heating rate of 5 °C/min from −50 °C to 150 °C in tensile mode with a frequency of 1 Hz. Differential scanning calorimetry (DSC) was conducted using a TA Q2000 to characterize the PUA monomers, with the first temperature scan from −80 °C to 100 °C at a heating rate of 20 °C/min. The crosslinked PUAs were characterized with the first temperature scan from −50 °C to 200 °C at a heating rate of 10 °C/min. Tensile testing was performed using an Instron 3365 at 25 °C, with a dumbbell-shaped spline having a width of 0.5 mm, a thickness of 1 mm in the middle section, and a testing speed of 20 mm/min.

### 3.3. Evaluation of Chemical Resistance

Gas chromatography (GC, Shimadzu GC 2010) was used for the retention of DMMP and CEES in the PUAs. The GC column was a DB-WAX (30 m length, 0.250 mm inner diameter, 0.25 μm film thickness) from Agilent. Temperature program: the starting temperature was 50 °C, held for 3 min, increased to 160 °C at a rate of 10 °C/min, and then increased to 200 °C at a rate of 40 °C/min, and held for 2 min. Injection volume: 1 μL; detector: FID detector. The standard curves of DMMP and CEES and the error assessment have been demonstrated in our previous studies [13]. The pretreatment for GC was as follows: DMMP or CEES (20 mg) was dropped uniformly on the surface of PUAs (2 cm × 2 cm) at 25 °C and 30–50% relative humidity. After different exposure times, the remaining agents on the surface were wiped, and then the PUAs were soaked in DCM for ultrasonic dispersion for 30 min; the extracted DCM extraction liquid was analyzed by GC to determine the amount of the agents. The retention of DMMP or CEES in the PUAs was calculated using Equation (1) [12,13,28].

(1) $$\mathrm{retention}\;(\%)=\frac{W_{r}}{W_{t}}\;\times 100\%$$

where *W*_t_ is the total mass of agents (mg) dropped at first, and *W*_r_ is the mass of agents (mg) absorbed (obtained from GC).

## 4. Results and Discussion

### 4.1. Composition and Chemical Structure of the PUAs

**Figure 2:**
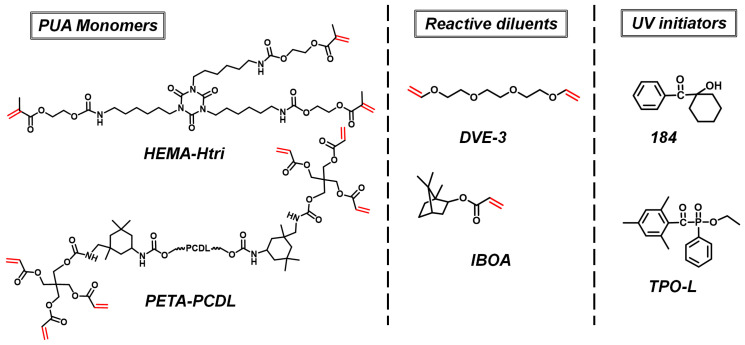
Chemical structures of PUA monomers, reactive diluents, and UV initiators.

The chemical structures of the two PUA monomers are shown in Figure 2, and the characterization of these monomers is detailed in the Supporting Information. The presence of the PUA monomer C=C was demonstrated by the appearance of two characteristic peaks of the protons (-C=C**H**_2_) linked to the double bond at a chemical shift of 6 ppm on the ^1^H NMR spectrum of Figure S1 and characteristic double peaks of the stretching vibration of C=C at 1635–1600 cm^−1^ on the FTIR spectrum of Figure S2, which indicated that C=C has not thermal polymerized. The absence of a -NCO (2250 cm^−1^) peak in the FTIR spectrum (Figure S2) indicates the complete reaction of two PUA monomers. The DSC curves of the two PUA monomers are shown in Figure S3. The *T_g_* measured by DSC of the PETA-PCDL monomer was −28.7 °C (Figure S3). Due to the high viscosity of these two PUA monomers, it is essential to incorporate reactive diluents into the PUA formulation. The reactive diluent can also participate in the curing reaction. In this paper, the less viscous IBOA (an acrylate) and DVE-3 (a vinyl ether) were used, and their chemical structures are shown in Figure 2. Taking HEMA-Htri as the resin and DVE-3 as the reactive diluent as an example (Figure S4), the viscosity of the monomer mixture reached 8425 cP when no reactive diluent was added, and it was dropped to 257 cP with the addition of 30 wt.% reactive diluent, which is favorable for the painting and the addition of other components. This kind of UV initiator has a great influence on both the initiation efficiency and resin properties after being cured. The free radical initiators 184 and TPO-L (2 wt.% of the total resin) used in this paper are known for their high efficiency and effectiveness in preventing yellowing of the UV-cured resins. Their chemical structures are shown in Figure 2. The wavelength range (370 nm of TPO-L and 325 nm of 184 are the peak absorption wavelengths) and the decomposition reaction of the initiators are depicted in Figure S5, respectively.

**Figure 3:**
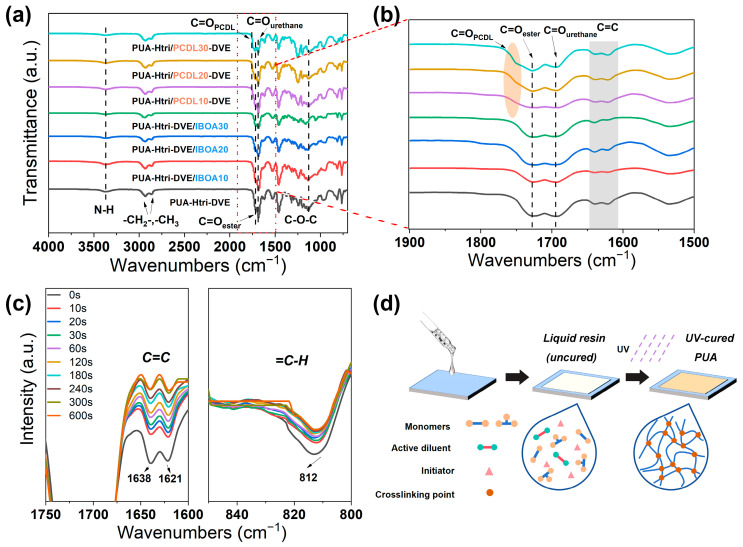
(**a**) FTIR spectra and (**b**) magnified spectra from 1900 to 1500 cm^−1^ of PUAs; (**c**) FTIR dynamic spectra of PUA-Htri-DVE with UV irradiation time. (**d**) Schematic diagram of PUA preparation.

In this paper, two PUA series were investigated. One series involved varying the ratio of the reactive diluents IBOA and DVE-3 (PUA-Htri-DVE/IBOA*m*, with *m* representing the mass percentage of IBOA of the total resin) in order to investigate the effect of the type and amount of the acrylate diluent and the vinyl ether diluent on the properties of PUAs. The other involved varying the ratios of the PUA monomers HEMA-Htri and PETA-PCDL (PUA-Htri/PCDL*n*, with *n* representing the mass percentage of PETA-PCDL of the total resin) in order to investigate the effect of the type and amount of HEMA-Htri and PETA-PCDL on the properties of PUAs. The formulation was designed in this way to investigate the effect of introducing IBOA (to increase the glass transition temperature of PUAs) and PETA-PCDL (to introduce the PCDL chain and increase crosslinking density) on the structure and chemical resistance properties of PUAs. Figure 3a shows the FTIR spectra of the UV-cured PUAs, and Figure 3b presents a zoomed-in view of their carbonyl (C=O) region, with C=O linked to the acrylate at 1720 cm^−1^, C=O linked to the urethane at 1690 cm^−1^, and C=O of the ester on the polycarbonate chain at 1750 cm^−1^. The vibrational peaks of the C=C stretching vibration at 1638 cm^−1^ and 1621 cm^−1^ and the vibrational peak of =C-H linked to C=C at 812 cm^−1^ appear in Figure 3b. Dynamic FTIR of PUA-Htri-DVE was measured with different UV irradiation times (Figure 3c), and the results demonstrate that C=C was able to react sufficiently (more than 95% conversion) within the first 5 min. Figure S6 shows the absorbance of the dynamic FTIR spectra. The approximate C=C conversion of PUA-Htri-DVE was calculated using Equation S1 in the Supporting Information. Figure 3d shows a schematic representation of the PUA coating preparation, indicating the transformation of the small molecules into a crosslinked network after UV irradiation. The obtained UV-cured PUAs were transparent (Figure S7).

### 4.2. Thermodynamic Properties of the PUAs

**Figure 4:**
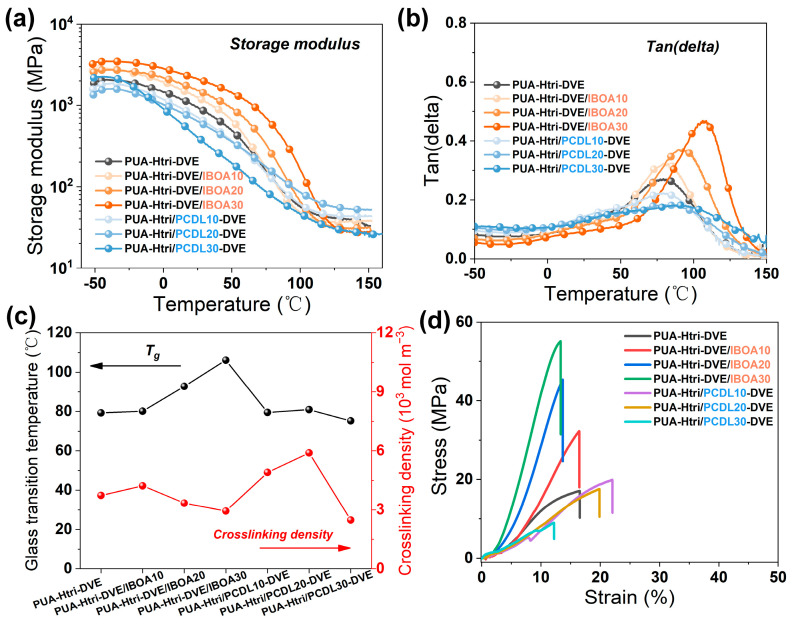
(**a**) Storage modulus and (**b**) loss angle tangent of the PUAs; (**c**) *T_g_* and crosslinking density of the PUAs; (**d**) stress–strain curve of the PUAs.

The storage modulus and loss angle tangent of PUAs are shown in Figure 4a,b, respectively. The peak temperature of the loss angle tangent is taken as *T_g_*. The crosslinking density (*υ_e_*) was calculated by Equation (2) with the storage modulus of the rubbery plateau obtained by DMA [29,30,31].

(2) $$\upsilon_{e}=\frac{E_{r}}{3RT}$$

where *E_r_* is the storage modulus of the rubbery plateau, Pa; R is the gas constant; *T* is taken as *T_g_* + 30, K.

The crosslinking density (*υ_e_*) calculated from Equation (2) is shown in Figure 4c and Table 2. As IBOA partially replaces DVE-3, the *T_g_* of the PUAs increases due to the site-blocking effect of the isobornyl ester, and the *T_g_* of PUAs (PUA-Htri-DV3/IBOA30) reaches 106 °C when IBOA was used as the unique reactive diluent. However, due to the replacement of bi-functional DVE-3 by mono-functional IBOA, there was a certain decrease in the *υ_e_*. For the PUA series containing the PCDL chain, the *T_g_* of the PUAs did not decrease with the increase in PCDL chains. The presence of the tri-functional hydroxypropyl acrylate (PETA) and the potential hydrogen bonding interaction between the ester bonds of PCDL chain segments and urea bonds can increase the *T_g_*. However, due to the flexibility of the PCDL chain segments, the final *T_g_* of the PUA remains essentially unchanged. The *υ_e_* of PUA-Htri/PCDL10-DVE and PUA-Htri/PCDL20-DVE increased due to the introduction of C=C hexafunctionality of PETA at both ends. However, the *υ_e_* of the PUA-Htri/PCDL30-DVE began to decrease with the amount of PCDL chain, which is probably due to the poor compatibility of the PCDL chain segments with the polyacrylate crosslinked network (The peak shape of Tan(delta) became flatter), which may have led to heterogeneity in the crosslinked network of PUA. Meanwhile, it was found the presence of PCDL chain addition might lead to incomplete curing of PUA, but no incomplete curing peaks were evident for PUA with IBOA. (See the DSC curves of the crosslinked PUAs in Figure S8). Figure 4d displays the stress–strain curves of PUAs. The tensile strength and elongation at break of PUA-Htri-DVE are 17.1 MPa and 17%, respectively. With the increase in IBOA, the tensile strength of PUA-Htri-DVE/IBOA-30 increased significantly up to 55.1 MPa, but its elongation at break is only 13%. The elongation at break of PUAs increased slightly with a small amount of PCDL chains; the elongation at break of Htri/PCDL10-DVE and PUA Htri/PCDL20-DVE are 20% and 22%, respectively. However, the mechanical properties decreased significantly when a large amount of PCDL chains were added; the tensile strength and elongation at break of PUA Htri/PCDL30-DVE are only 9.0 MPa and 12.2%, respectively.

### 4.3. Chemical Resistances of the PUAs

**Figure 5:**
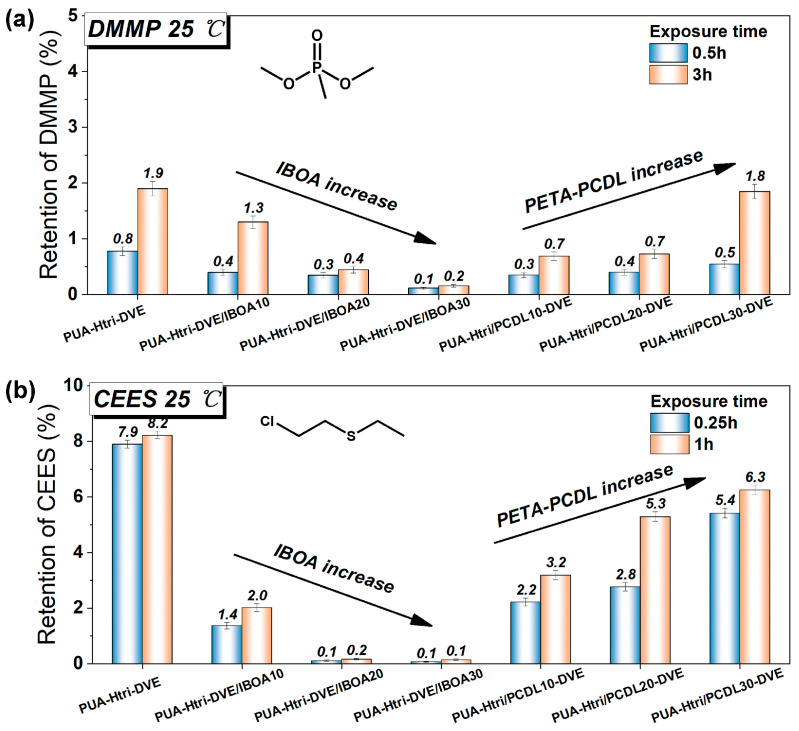
Retention of PUAs to (**a**) DMMP and (**b**) CEES calculated from GC data.

The assessment of the chemical protection properties of PUAs was explored using gas chromatography (GC) by detecting the retention of the two CWA simulants in PUA coatings. The GC process is shown in Figure S9, and the standard curves for the two CWA simulants are shown in Figure S10. Dimethyl methylphosphonate (DMMP) is a simulant for organophosphorus nerve agents, and 2-chloroethyl ethyl sulfide (CEES) is a simulant for the vesicant agent mustard. Our previous studies found that the permeation and interaction of these two on polymer coatings were large and varied. As for the barrier to DMMP (Figure 5a), the PUAs all showed good barrier performance (less than 2% retention). The retention rate decreased with increasing IBOA, and the 3 h retention rate of PUA-Htri-DVE/IBOA30 was less than 0.2%. Compared with PUA-Htri-DVE, the small addition of the PCDL chain can reduce a certain retention rate of DMMP. The phosphate ester (P=O) in DMMP is more polar and has a strong permeability to the polymer, so the addition of a large amount of long-chain polyol (e.g., PCDL) reduces the barrier performance of the crosslinked network to small molecules. As for the barrier to CEES (Figure 5b), PUA-Htri-DVE had poor protection against CEES with a retention of 7.9% at 1 h, but the addition of IBOA significantly reduced the retention of CEES and improved the protection. The retention of CEES by PUA-Htri-DVE/IBOA30 was less than 0.1%. IBOA increased the *T_g_* of PUA and also the resistance to CEES. The addition of PCDL chains decreased the retention of CEES, but the retention increased with the amount of PCDL chains, which may be due to the flexibility of PCDL chains leading to its more permeation of CEES. Specific values for retention rates are shown in Table 3. The possible interactions of the polyurethane chemical structure with DMMP and CEES are shown in Figure S11. The *T_g_* value of both PUA-Htri-DVE/IBOA20 (*T_g_* = 92.8 °C) and PUA-Htri-DVE/IBOA30 (*T_g_* = 106.1 °C) was significantly higher than that of PUA-Htri-DVE (79.3 °C), which enhanced the properties of the crosslinked network of PUAs in the glassy state, thus enhanced the resistance to DMMP and CEES. This may be indicated by the fact that glassy crosslinked PUA networks with high *T_g_* have better resistance to both DMMP and CEES. Compared to PUA-Htri-DVE (*υ_e_* = 3439 mol·m^−3^), although the crosslinking density of PUA-Htri/PCDL10-DVE (*υ_e_* = 4516 mol·m^−3^) and PUA-Htri/PCDL20-DVE (*υ_e_* = 5429 mol·m^−3^) significantly increased, they did not show a noticeable improvement in resistance to DMMP and CEES, which may be attributed to the fact that the crosslinking density of the PUA has already reached a certain level. This may also indicate that the flexible PCDL chain regions are susceptible to chemical permeation into the crosslinked network. From the peak shape of Tan(delta) in Figure 4b, it seems that the addition of PCDL chains (broadening and flattening of the Tan(delta) peak) produces a certain heterogeneity in the highly crosslinked network of the acrylates, which may affect the improvement in the chemical protection of the PUAs.

## 5. Conclusions

In summary, seven UV-cured, highly crosslinked PUA coatings were prepared using HEMA-Htri and PETA-PCDL as monomers, with IBOA and DVE-3 serving as reactive diluents. As the addition of IBOA increased, *T_g_* of PUA rose, tensile strength increased, and protection against DMMP and CEES improved significantly. These findings suggest that both *T_g_* and *υ_e_* are critical factors influencing the chemical protection performance of PUAs. PUA-Htri-DVE/IBOA20 and PUA-Htri-DVE/IBOA30 demonstrated excellent protection performance against DMMP and CEES (both retentions are less than 0.5%). The addition of PETA-PCDL segments to enhance PUA protection was found to be unsatisfactory. Excessive PCDL addition resulted in a decrease in crosslinking density. Moreover, the polyol PCDL chain might serve as a vulnerable site for attack by DMMP and CEES. These studies could prove valuable for developing highly chemically resistant coatings.

## Figures and Tables

**Table 1:** Formulations of the PUA coatings.

Formula/wt.%	PUA Monomer		Reactive Diluent		UV Initiator	
	HEMA-Htri	PETA-PCDL	IBOA	DVE-3	TPO-L	184
PUA-Htri-DVE	68.63			29.41	1.57	0.39
PUA-Htri-DVE/IBOA10	68.63		9.80	19.61	1.57	0.39
PUA-Htri-DVE/IBOA20	68.63		19.61	9.80	1.57	0.39
PUA-Htri-DVE/IBOA30	68.63		29.41		1.57	0.39
PUA-Htri/PCDL10-DVE	58.83	9.80		29.41	1.57	0.39
PUA-Htri/PCDL20-DVE	49.02	19.61		29.41	1.57	0.39
PUA-Htri/PCDL30-DVE	39.22	29.41		29.41	1.57	0.39

**Table 2:** The crosslink density of the PUs calculated from DMA.

Samples	*T_g_*/°C	Storage Modulus at *T_g_*/MPa	Storage Modulus at *T_rubbery plateau_*/MPa	*υ_e_*/mol·m^−3^
PUA-Htri-DVE	79.3	129.2	32.8	3439
PUA-Htri-DVE/IBOA10	80.1	123.5	37.2	3892
PUA-Htri-DVE/IBOA20	92.8	104.1	30.5	3088
PUA-Htri-DVE/IBOA30	106.1	99.1	27.8	2724
PUA-Htri/PCDL10-DVE	79.5	123.2	43.1	4516
PUA-Htri/PCDL20-DVE	80.9	146.5	52.0	5429
PUA-Htri/PCDL30-DVE	80.7	74.9	26.6	3013

**Table 3:** Retention of PUAs to DMMP and CEES in this paper.

Samples	Retention/%			
	DMMP/0.5 h	DMMP/3 h	CEES/0.25 h	CEES/1 h
PUA-Htri-DVE	0.78 ± 0.08	1.90 ± 0.13	7.90 ± 0.14	8.23 ± 0.14
PUA-Htri-DVE/IBOA10	0.40 ± 0.05	1.30 ± 0.11	1.37 ± 0.12	2.02 ± 0.14
PUA-Htri-DVE/IBOA20	0.34 ± 0.05	0.44 ± 0.06	0.12 ± 0.02	0.17 ± 0.03
PUA-Htri-DVE/IBOA30	0.11 ± 0.02	0.16 ± 0.03	0.08 ± 0.02	0.15 ± 0.03
PUA-Htri/PCDL10-DVE	0.35 ± 0.05	0.69 ± 0.07	2.22 ± 0.15	3.19 ± 0.17
PUA-Htri/PCDL20-DVE	0.40 ± 0.05	0.73 ± 0.08	2.77 ± 0.16	5.30 ± 0.17
PUA-Htri/PCDL30-DVE	0.55 ± 0.06	1.85 ± 0.13	5.42 ± 0.17	6.26 ± 0.17

## Data Availability

Data is contained within the article or Supplementary Material.

## Supplementary Materials

The following supporting information can be downloaded at https://www.mdpi.com/article/10.3390/polym16111578/s1. Figure S1: ^1^H NMR spectra of (a) HEMA-Htri and (b) PETA-PCDL; Figure S2: FTIR spectra of HEMA-Htri and PETA-PCDL; Figure S3: DSC curves of the HEMA-Htri and PETA-PCDL; Figure S4: The relationship between different reactive diluent additions and the resin viscosity (resin: HEMA-Htri, reactive diluent: DVE-3); Figure S5: UV initiation wavelength range and decomposition reaction of the UV initiators (184 and TPO-L); Figure S6: Absorbance spectra of the dynamic FTIR spectra; Figure S7: Digital photographs of the transmittance of the PUAs; Figure S8: DSC curves of the crosslinked PUAs; Figure S9: Procedures for assessing chemical defence performance in our research; Figure S10: GC standard curves of DMMP and CEES; Figure S11: Possible interactions of PU chemical structure with DMMP and CEES. Supporting information is available, with the additional characterization of the two PUA monomers and the PUAs. Reference [32] is cited in the supplementary materials.
