# Supplementary material for: UV-Cured Highly Crosslinked Polyurethane Acrylate to Serve as a Barrier against Chemical Warfare Agent Simulants
Source: Polymers (Basel). 2024 Jun 2;16(11):1578. doi: 10.3390/polym16111578 (PMC11175127; doi:10.3390/polym16111578)
Supplement: Supplementary file 1 [file polymers-16-01578-s001.zip › polymers-3009426-supplementary.pdf]

Supporting Information

UV Cured Highly Crosslinked Polyurethane Acrylate to Barrier Against Chemical Warfare Agent Simulants

Xucong Chen,[1,2] Linjing Xiao,[2] Hong Li,*[,2] Yan Cui,*[,3] and Guiyou Wang*[,1]

[1] *Shanghai Key Laboratory of Advanced Polymeric Materials, School of Materials Science and Engineering, East China University of Science and Technology, Shanghai 200237, China*

[2] *School of Chemistry and Chemical Engineering, Shanghai Key Laboratory of Electrical Insulation and Thermal Aging, Shanghai Jiao Tong University, Shanghai 200240, China*

[3] *State Key Laboratory of NBC Protection for Civilian, Beijing 102205, China*

***Email:**

Hong Li: lh102@sjtu.edu.cn*

Guiyou Wang: guiyouwang@ecust.edu.cn*

Yan Cui: tracypiscescy@163.com*

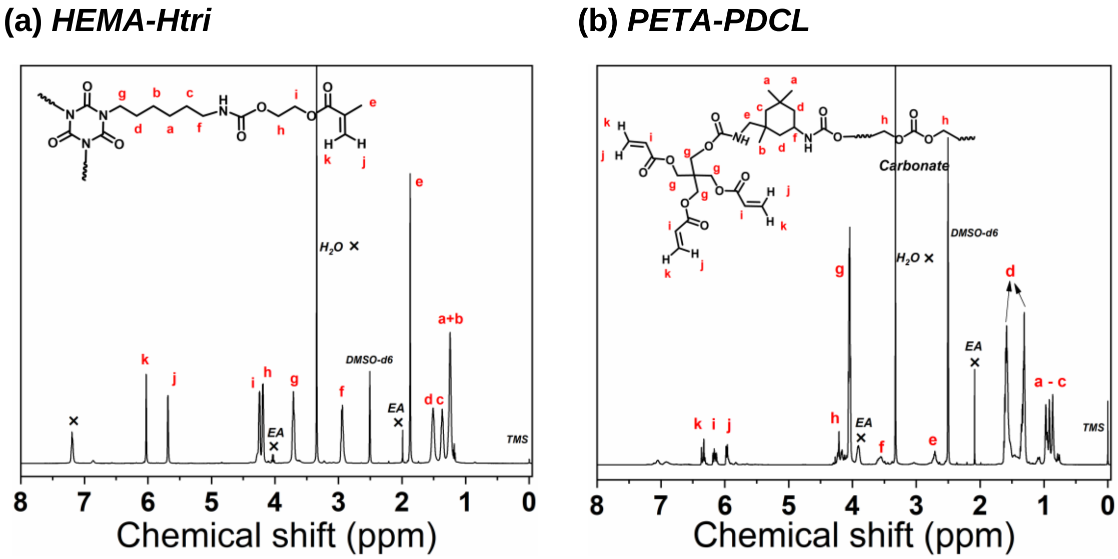

Figure S1 ^{1}H NMR spectra of (a) HEMA-Htri and (b) PETA-PCDL

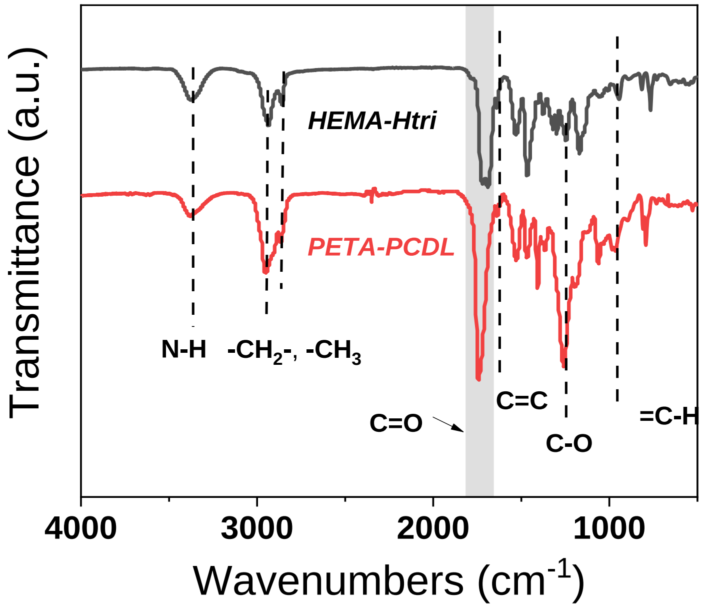

Figure S2 FTIR spectra of the HEMA-Htri and PETA-PCDL

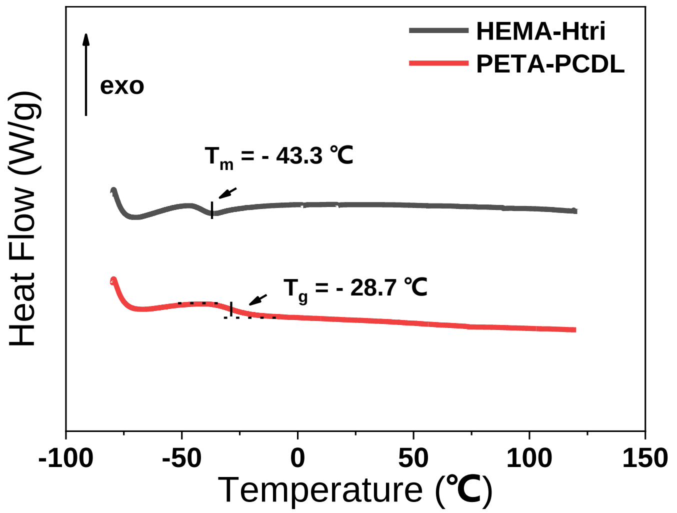

Figure S3 DSC curves of the HEMA-Htri and PETA-PCDL

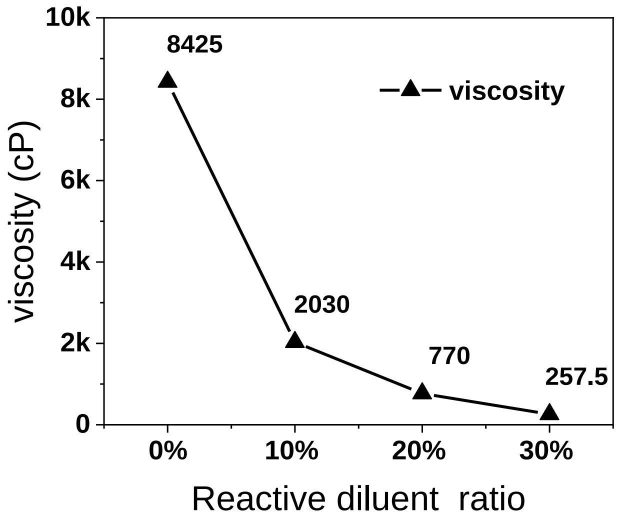

Figure S4 The relationship between different reactive diluent additions and the resin viscosity (resin: HEMA-Htri, reactive diluent: DVE-3)

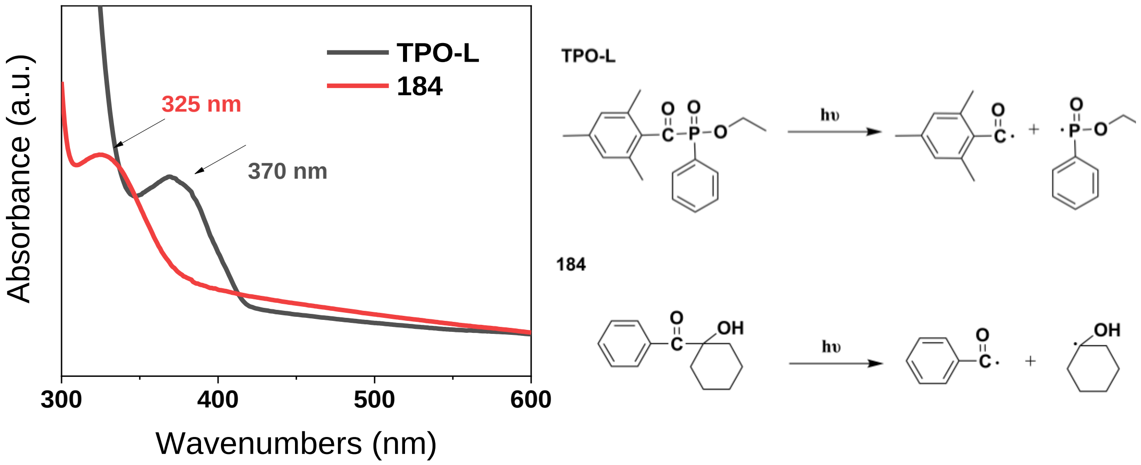

Figure S5 UV initiation wavelength range and decomposition reaction of the UV initiators (184 and TPO-L)

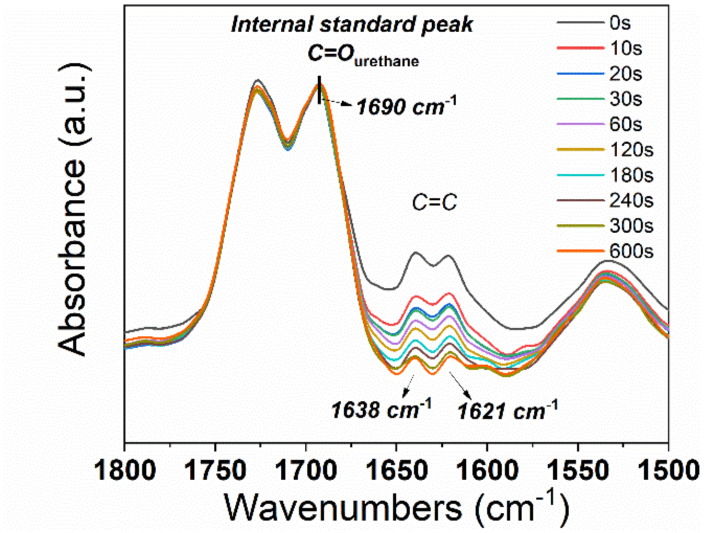

Figure S6 Absorbance spectra of the dynamic FTIR spectra

$$\text{C=C conversion} = \frac{\left(\frac{A_{1638}+A_{1621}}{A_{1690}}\right)_0 - \left(\frac{A_{1638}+A_{1621}}{A_{1690}}\right)_t}{\left(\frac{A_{1638}+A_{1621}}{A_{1690}}\right)_0 - \left(\frac{A_{1638}+A_{1621}}{A_{1690}}\right)_c} \times 100\% \qquad \textbf{Equation S1}$$

Where A_{1638} and A_{1621} is the absorbance of the C=C peak and A_{1690} is the absorbance of the $C{=}O_{urethane}$ peak, A_0 refers to the absorbance at the moment 0, A_t refers to the absorbance at the moment t, A_c refers to the absorbance after PUA complete curing.[1]

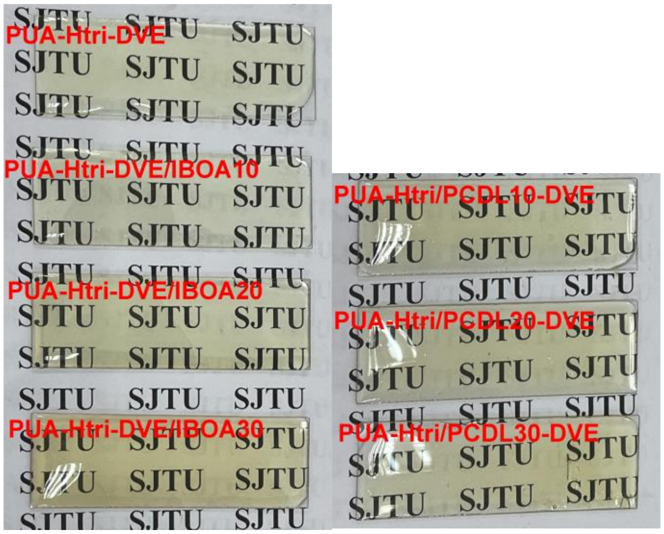

Figure S7 Digital photographs of the transmittance of PUAs

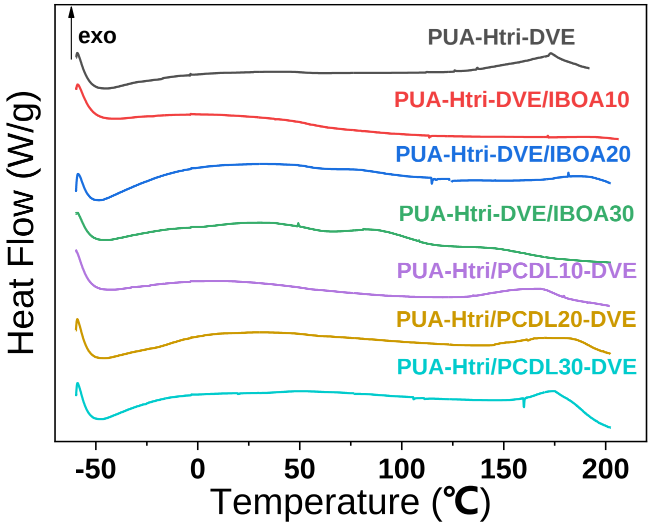

Figure S8 DSC curves of the crosslinked PUAs

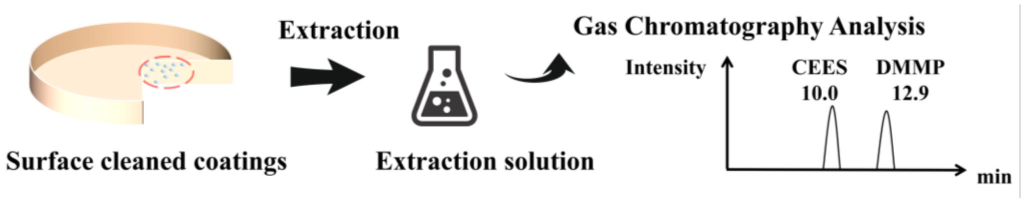

Figure S9 Procedures for assessing chemical defence performance in our research [2,3]

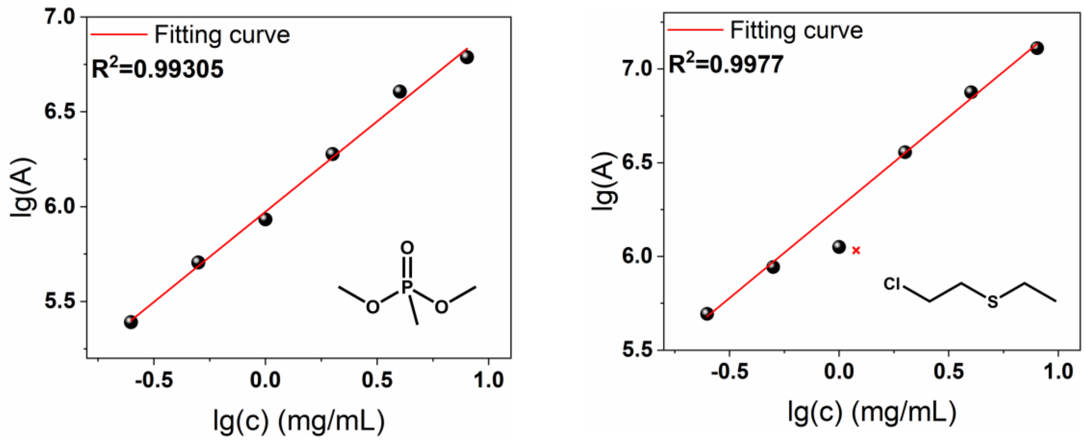

Figure S10 GC standard curves of DMMP and CEES [2,3]

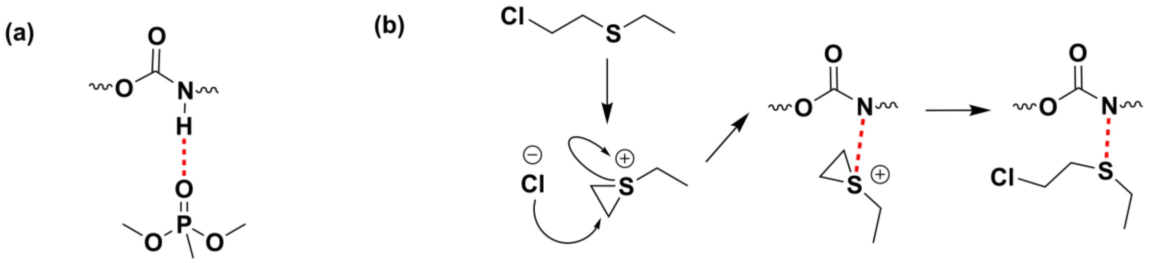

Figure S11 Possible interactions of PU chemical structure with DMMP and CEES

References

1. Huang, X.; Peng, S.; Zheng, L.; Zhuo, D.; Wu, L.; Weng, Z. 3D Printing of High Viscosity UV-Curable Resin for Highly Stretchable and Resilient Elastomer. *Adv. Mater.* **2023**, *35*, e2304430, doi:10.1002/adma.202304430.
2. Chen, X.; Li, X.; Zhang, H.; Li, H.; Wang, G.; Cui, Y. Cross-Linked Fluorinated Polyurethanes against Chemical Warfare Agent Simulants: Properties Affected by the Structure of Diisocyanate. *Macromolecules* **2023**, *56*, 8993-9002, doi:10.1021/acs.macromol.3c01489.
3. Wu, G.; Zhang, D.; Xu, W.; Zhang, H.; Chen, L.; Zheng, Y.; Xin, Y.; Li, H.; Cui, Y. Highly Cross-linked Epoxy Coating for Barring Organophosphate Chemical Warfare Agent Permeation. *ACS Omega* **2022**, *7*, 12354-12364, doi:10.1021/acsomega.2c00915.
